# Subarctic-scale transport of ^134^Cs to ocean surface off northeastern Japan in 2020

**DOI:** 10.1038/s41598-023-34775-8

**Published:** 2023-05-09

**Authors:** Mutsuo Inoue, Kaisei Mashita, Hiroaki Kameyama, Hayata Mitsunushi, Yota Hatakeyama, Yukiko Taniuchi, Takuya Nakanowatari, Takami Morita, Seiya Nagao

**Affiliations:** 1 Low Level Radioactivity Laboratory, Kanazawa University, O-24, Nomi, Ishikawa 923-1224 Japan; 2 Fisheries Resources Institute, 116 Katsurakoi, Kushiro, Hokkaido 085-0805 Japan; 3 Fisheries Resources Institute, 2-12-4, Fukuura, Kanazawa, Yokohama 236-8648 Japan

**Keywords:** Environmental sciences, Ocean sciences

## Abstract

We studied the spatiotemporal variations in ^134^Cs, ^137^Cs, and ^228^Ra concentrations at the sea surface off southeastern Hokkaido, Japan (off-Doto region) from 2018 to 2022 using low-background γ-spectrometry. The ^134^Cs concentrations in the off-Doto region, decay-corrected to the date of the Fukushima Dai-ichi Nuclear Power Plant (FDNPP) accident, exhibited wide lateral variation each year (e.g., 0.7–1.1 mBq/L in 2020). By studying the ^228^Ra concentrations and salinity, this variation was explained based on the current mixing patterns. Furthermore, the ^134^Cs concentrations in the waters highly affected by the Oyashio Current (OYC) gradually increased from 2018 to 2020, and subsequently decreased in 2022. This implies that the water mass maximally contaminated with ^134^Cs was transported back to the side of the Japanese islands 10 years after the FDNPP accident along with counter-clockwise currents (e.g., the OYC) in the northern North Pacific Ocean. The ^134^Cs concentrations in the OYC-affected waters in the off-Doto region in 2020 were ~ 1/6 times those in the ^134^Cs-enriched core of waters off the western American Coast in 2015, which can be ascribed to dilution via spatial dispersion during subarctic current circulation. Overall, we elucidated the ocean-scale subarctic current systems in the northwestern North Pacific Ocean, including water circulation timespans.

## Introduction

The Fukushima Dai-ichi Nuclear Power Plant (FDNPP) accident, which occurred on March 11, 2011, led to the release of large amounts of radiocesium (^134^Cs and ^137^Cs) into the northwestern North Pacific Ocean, particularly around eastern Japan^1^. While the ^137^Cs (half-life: 30.2 years) content in seawater samples examined in this study is affected by a remnant of the global fallout from atmospheric nuclear test explosions (particularly from the mid-1950s to early 1960s), the detected ^134^Cs is believed to have entirely originated from the FDNPP accident owing to its shorter half-life (2.06 years). Because the time of introduction of ^134^Cs (March 2011) into the seawater, areas of direct discharge into the seawater (close to the FDNPP), and radioactive deposition behavior of ^134^Cs (the northwestern North Pacific Ocean) are known^2,3^, this radionuclide has emerged as a strong chemical tracer of water circulation; the circulation can be tracked until the radionuclide is undetectable owing to its radioactive decay and dispersion. In the subtropical area, low levels of ^134^Cs were carried back to the side of the Japan islands (to the Sea of Okhotsk via the Sea of Japan) from 2013 by the clockwise Kuroshio Warm Current (KWC)^4,5^. In contrast, ^134^Cs was also transported to the western Bering Sea until 2018 in the subarctic area via the western American Coast^6–9^. Furthermore, our previous radionuclide studies conducted off southeastern Hokkaido, Japan (hereinafter referred to as off-Doto) in 2018 and 2019 indicated that ^134^Cs was transported to the off-Doto region by the Oyashio Current (OYC) via the East Kamchatka Current (EKC), with contributions from other currents around Hokkaido^10^.

Additionally, the lateral distributions of the concentrations of ^228^Ra—a natural and soluble radionuclide with a half-life of 5.75 years and a conventional tracer used for studying water currents^11,12^—have been employed to study the transport patterns of radiocesium in the seas around Japan^13^.

In this study, we used special low-background γ-spectrometry to precisely examine temporal and lateral variations in low-level ^134^Cs, ^137^Cs, ^226^Ra, and ^228^Ra concentrations in the surface waters in and around the off-Doto region during 2020–2022. The ^226^Ra concentrations in the water samples, which are useful for understanding vertical circulations, will be presented elsewhere. Furthermore, by focusing on ^134^Cs concentrations, we discussed the ocean-scale subarctic current systems in the northwestern North Pacific Ocean, including the timescale, after the FDNPP accident. This study can, therefore, provide a basis for predicting the transport patterns of soluble contaminants in the waters.

## Results

### Current systems from ^228^Ra–salinity

**Figure 1:**
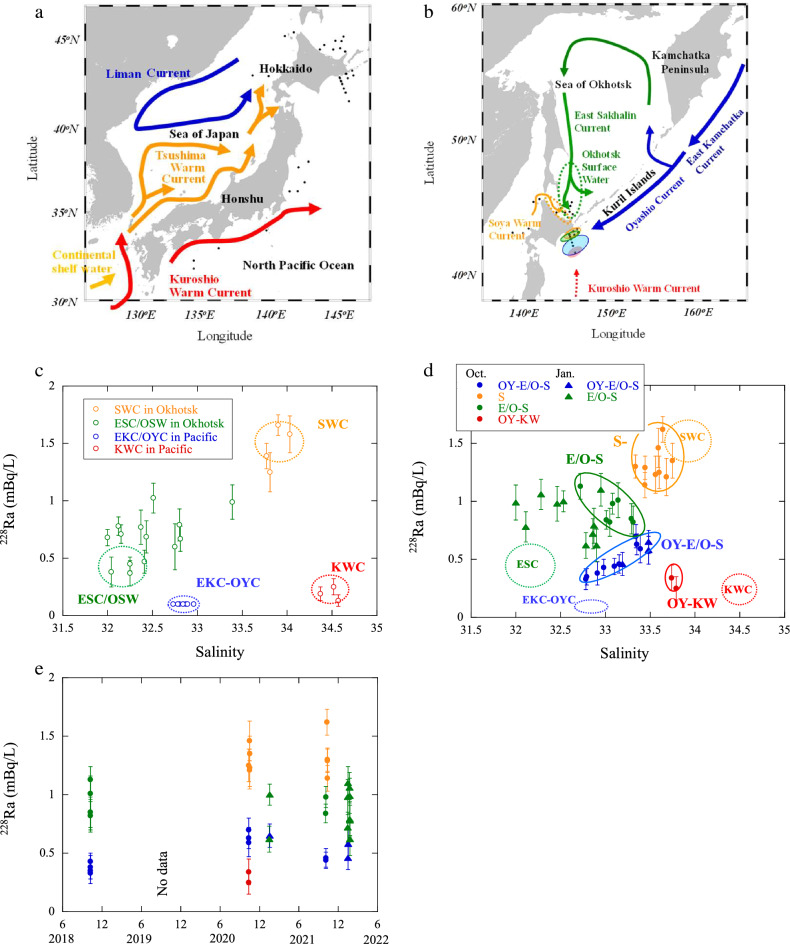
(**a**) Seawater sampling locations around the Honshu and (**b**) Hokkaido islands in Japan, along with the major current systems^14–16^. ^228^Ra concentrations *vs.* salinity in the source currents to the off-Doto region in (**c**) the adjacent sea areas and (**d**) the off-Doto region, with the data areas of in the source currents, and (**e**) temporal variation in ^228^Ra concentrations at the sea surface in the off-Doto region during 2018–2022. Data regarding the SWC, ESC/OSW, OYC, and off-Doto waters in 2018 are partially from previous reports^10^.

Major ocean current systems in and around the off-Doto region are shown in Fig. 1a,b^14–16^. The subarctic EKC extends along the Kuril Islands and reaches the off-Doto region as the OYC. A branch of the OYC partially enters the Sea of Okhotsk, and after a counter-clockwise circulation, returns as a southward current along the Sakhalin Island as the East Sakhalin Current (ESC). The Tsushima Warm Current (TWC), mainly composed of the KWC, flows from the Sea of Japan into the Sea of Okhotsk and circulates along the northeastern coast of Hokkaido as the Soya Warm Current (SWC).

The sea surface in the off-Doto region is predominantly composed of a mixture of the SWC, ESC, and Okhotsk Sea Surface Water (OSW) from the southwestern Sea of Okhotsk and the westward OYC. In contrast, in the south of the off-Doto region, there are occasional intrusions by a warm-core ring that separates from the subtropical KWC.

The γ-spectrometry results are shown in Table S1. To elucidate the mixing pattern of the coexisting currents, ^228^Ra concentrations at the sea surfaces adjacent to and in the off-Doto region were plotted against salinity, as shown in Fig. 1c,d, respectively. Salinity at the surface of the subtropical SWC and KWC is significantly higher than that of the subarctic ESC/OSW and OYC (33.4–34.5 and 32.4–33.4, respectively)^10,17^. Notably, the ^228^Ra concentrations in the SWC are the highest among the currents in this study area. This is because ^228^Ra is transported by the TWC from the Sea of Japan, which receives extensive supply from the shallow shelf in the western East China Sea^12^. The major source currents to the sea surface in the off-Doto region are the high-salinity and highest-^228^Ra SWC, the lowest-salinity ESC/OSW, and lowest-^228^Ra EKC–OYC^8,18^ (Fig. 1c).

The surface waters in the off-Doto region have been classified into highly SWC-, ESC/OSW–SWC-, and OYC–ESC/OSW–SWC-affected seawaters (hereafter, the S-, E/O–S-, and OY–E/O–S-waters), as can be seen in Fig. 1d, neglecting the ambiguous supplies of ^228^Ra after passing through the Soya Strait. Notably, the high salinity and low ^228^Ra concentrations of the waters in the southern off-Doto region (< 42° N) in 2020 indicated the contribution of the KWC, which had the highest salinity among the currents studied. The waters are therefore considered highly affected by OYC and KWC (OY–KW-waters). Because of the absence of ^228^Ra data, samples collected in the off-Doto region in 2019^10^ and a sample in 2021 have been tentatively classified as the S- (salinity: > 33.4), E/O–S- or OY–E/O–S- (salinity: 33.4–34.3), and OY–KW-waters (salinity: ~ 34.5), based on their salinity and sampling areas. The fractions of current sources and the mixing patterns at the sea surface in the off-Doto region showed year to year variation during 2018–2022 (e.g., the large contribution of the S-waters in October 2020 and 2021) (Fig. 1e). The coastal area in the off-Doto region is predominantly occupied by the S- and E/O–S-waters from the southwestern Sea of Okhotsk, while the sea surface in the offshore area is often composed of the OY–E/O–S-waters. Furthermore, the current system in the off-Doto region exhibited seasonal variation—for instance, the E/O–S-waters had lower salinity and higher ^228^Ra concentrations in January (Fig. 1d). The fraction of the low-salinity ESC/OSW is higher in January^16^, although ^228^Ra could be supplied from the coastal and lake sediments and/or a seasonal change in the mixing ratio of the ESC and OSW.

### Annual variations of radiocesium

**Figure 2:**
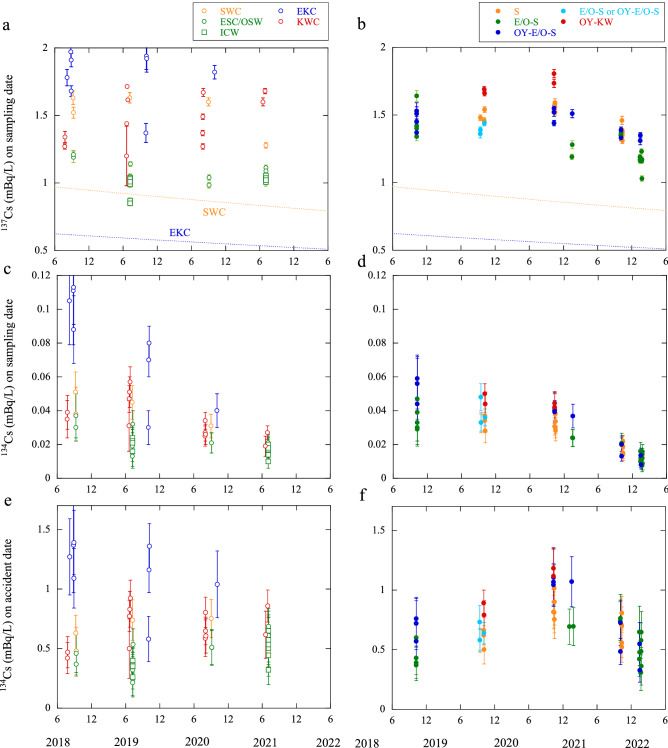
Temporal variations during 2018–2022: ^137^Cs concentrations at the sea areas (**a**) adjacent to and (**b**) in the off-Doto region decay-corrected to the sampling date, with the decay curves calculated based on the effective half-life (13.7 years)^19^ and considering 0.9 mBq/L for the EKC and 1.4 mBq/L for the SWC in March 2011, respectively^10^; ^134^Cs concentrations in the sea areas (**c**) adjacent to and (**d**) in the off-Doto region decay-corrected to the sampling date; and ^134^Cs concentrations (**e**) adjacent to and (**f**) in the off-Doto region decay-corrected to the date of the FDNPP accident. Data of the ICW, EKC, and off-Doto waters in 2018 and 2019 are from previous reports^8,10,21^.

The annual variations of ^134^Cs and ^137^Cs concentrations in and around the off-Doto region are shown in Fig. 2, along with the current definition for each sample based on the ^228^Ra concentrations and salinity (Fig. 1c,d).

The concentrations of the global fallout-derived ^137^Cs, decay-corrected to the sampling date, in the subarctic ESC/OSW are clearly lower than those in the subtropical SWC and KWC (Fig. 2a); the concentrations in the SWC, ESC, and EKC immediately before the FDNPP accident were estimated to be 1.4, 1.0, and 0.9 mBq/L, respectively^10^. The difference in ^137^Cs concentrations in the Sea of Okhotsk predominantly retained the original features of the SWC and ESC/OSW (concentration levels of the global fallout-derived ^137^Cs calculated using an effective half-life of 13.7 years^19^), with the addition of the FDNPP-derived ^137^Cs during 2018–2022. However, despite the subarctic current, the ^137^Cs concentrations in the EKC in and around the Kamchatka Strait were higher than those in the other currents^8,9^, reflecting larger contribution of the FDNPP-derived ^137^Cs.

The ^137^Cs concentrations at the surface in the off-Doto region exhibited lateral variations every year; the concentrations levels differed for each current type, reflecting the combination of global fallout- and FDNPP-derived ^137^Cs (Fig. 2b). Additionally, the ^137^Cs concentrations were highest in October 2020 (1.4–1.7 mBq/L), but subsequently decreased, as can be seen from the concentrations of the samples in October 2021. The ^137^Cs concentrations in the off-Doto region in October 2021 and January 2022 were lower than those in October 2020 and January 2021.

In contrast, owing to the short half-life of ^134^Cs, the ^134^Cs concentrations, decay-corrected to the sampling date, decreased from ~ 0.1 to ~ 0.01 mBq/L in the samples from the current source areas off-Doto and from ~ 0.06 to ~ 0.01 mBq/L in the samples from the off-Doto region during this period (Fig. 2c,d).

To simplify the comparison of the concentrations of FDNPP-derived radiocesium, we focused on the ^134^Cs concentrations, and eliminated the effect of radioactive decay by decay-correcting the concentrations to the date of the FDNPP accident based on the physical half-life (2.06 years) (Fig. 2e,f). In the surface seawaters of the western Bering Sea and EKC area, the ^134^Cs concentrations (decay-corrected to the accident date) exhibited a small variation (1–2 mBq/L) between 2018 and 2020 after an initial increase between 2013 and 2017 (0.5–1 mBq/L)^8,9^ (Fig. 2e). The ^134^Cs concentrations in the EKC showed the highest values among the source waters in the off-Doto region. This indicates that ^134^Cs was transported from the western Bering Sea, particularly from 2017.

The ^134^Cs concentrations in the subtropical SWC showed a small variation between 2018 and 2021, ranging from 0.5 to 0.8 mBq/L. Furthermore, the values were slightly lower than those in the KWC in the Pacific Ocean along the side of the Japan islands. This is probably because the KWC is diluted by the continental shelf water less contaminated with ^134^Cs from the western East China Sea and the subarctic currents off the western coast of Hokkaido in the northeastern Sea of Japan^20^.

In contrast, the ^134^Cs concentrations in the ESC/OSW in the southwestern Sea of Okhotsk, including the intermediate cold water (ICW; ~ 50–300 m in depth), continued to increase from 0.3–0.4 mBq/L in 2019 to 0.4–0.7 mBq/L in 2021, following an increase in concentration that previously occurred between 2013 and 2017^21^. This was ascribed to an increase in ^134^Cs concentrations in the EKC that entered the Sea of Okhotsk.

The wide lateral variations in ^134^Cs concentrations in the off-Doto region in both 2018 and 2019 were explained by the mixing patterns of the SWC, ESC/OSW, and OYC^10^. The ^134^Cs concentrations in the off-Doto region also exhibited wide variation each year during 2020–2022 (e.g., 0.8–1.2 mBq/L in 2020) (Fig. 2f), reflecting the circulation paths and concentration level in each current. Additionally, the ^134^Cs concentrations at the surface in the off-Doto region showed large annual variation; the mean concentrations gradually increased from 0.6 mBq/L in October 2018 to 0.7 mBq/L in October 2019, and then to 1.0 mBq/L in October 2020. Notably, the ^134^Cs concentrations of all the current systems were also higher in October 2020 (mean; OY–KW-waters, 1.2 mBq/L; OY–E/O–S-waters, 1.1 mBq/L; S-waters, 0.9 mBq/L) and January 2021 (OY–E/O–S-water, 1.1 mBq/L) than in other years. However, in contrast, the ^134^Cs concentrations decreased to a mean value of 0.7 mBq/L in October 2021. Furthermore, reflecting the different current patterns between October and January^16,22,23^, the ^134^Cs concentrations in January were lower than those in October. However, the concentrations in the seawaters also decreased from a mean of 0.8 mBq/L in January 2021 to 0.5 mBq/L in January 2022.

## Discussion

The ^137^Cs concentrations in the areas adjacent to the off-Doto region showed a wide variation, with particularly low values in the ESC/OSW in the southwestern Sea of Okhotsk, thereby retaining the features of the global fallout-derived concentrations (Fig. 2a). In contrast, the high ^137^Cs concentrations in the subarctic EKC can be attributed to the addition of the FDNPP-derived ^137^Cs. The variation in the off-Doto region is smaller, including the E/O–S-waters—which are highly affected by the ESC/OSW (Fig. 2b). This is ascribed to the addition of the FDNPP-derived ^137^Cs to the E/O–S-waters via the OYC in the off-Doto region.

Reflecting the seasonal variation of current patterns, the contribution of the ESC (and consequently, the OSW) to the surface in the off-Doto region is higher in January than in October^16^ (Fig. 1d). This explains the lower ^137^Cs concentrations in January 2021 and 2022 (Fig. 2b). The high ^137^Cs concentrations in the OY–KW-waters, on the other hand, are ascribed to the KWC, which comprises the high global fallout-derived ^137^Cs, and the addition of the FDNPP-derived ^137^Cs.

**Figure 3:**
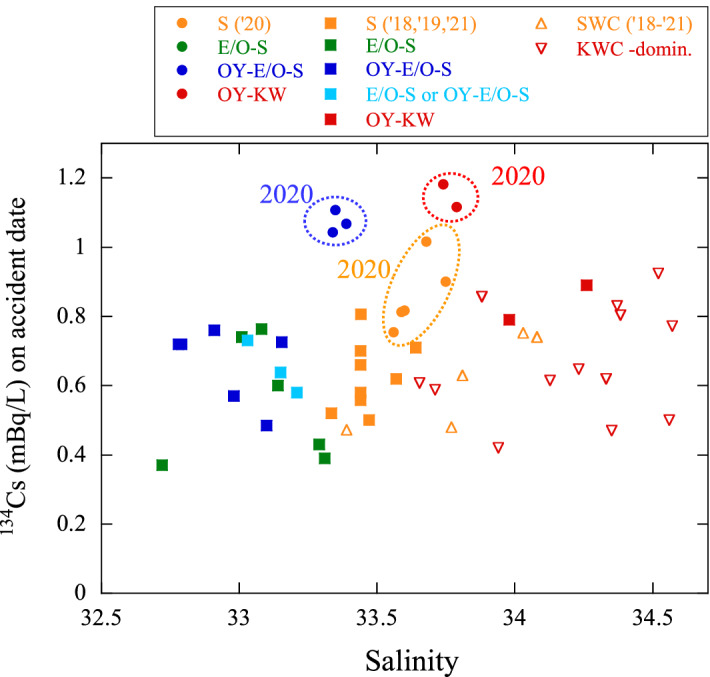
^134^Cs concentrations decay-corrected to the date of the FDNPP accident *vs.* salinity at the surface in the off-Doto region in October 2018–2021 along with the data of SWC- and KWC-dominated waters in mainly July 2018–2021.

The ^134^Cs concentrations of surface seawaters in the off-Doto region in October 2018–2021 are plotted against salinity in Fig. 3, along with the data of the KWC- and SWC-dominated waters. In 2020, the OY–KW-waters had the highest ^134^Cs concentrations in all currents and sampling period, along with higher salinity (and higher water temperature; Table S1) than in other samples collected from the Hokkaido side. From the lower ^134^Cs concentrations with small annual variations in the KWC- and SWC-dominated waters (Fig. 2e), it can be presumed that these subtropical currents did not contribute to the increase in ^134^Cs concentrations in the OY–KW-waters in 2020.

Notably, the ^134^Cs concentrations in the OY–E/O–S-waters in October 2020 (and January 2021) are significantly higher than those in other periods (Figs. 2f and 3). From the higher salinity and higher ^228^Ra concentrations among the OY–E/O–S-waters (Figs. 1d and 3), it is evident that the OY–E/O–S-waters in 2020 had a lower fraction of the EKC–OYC than in other years. Additionally, the salinity in S-waters in 2020 was higher than that in other years, indicating a lower fraction of the low-salinity EKC–OYC. The ^134^Cs concentrations in the OY–E/O–S- and S-waters subsequently decreased in October 2021. Additionally, the contribution of the FDNPP-derived ^137^Cs in the OY–E/O–S- and S-waters decreased steeply from 2020 to 2021, reflecting a decrease in the FDNPP-derived ^137^Cs in the current systems. Therefore, the highest ^134^Cs and ^137^Cs concentrations in the OY–KW- and OY–E/O–S-waters in 2020 can be predominantly attributed to the mixing of the OYC—which had the highest FDNPP-derived radiocesium concentrations in 2020—reaching the off-Doto region. The FDNPP-derived radiocesium concentrations in the off-Doto region reaching a maximum in 2020 implies that the subarctic ocean-scale current systems in the northern North Pacific Ocean have a timescale of ~ 10 years. Higher ^134^Cs concentrations in the S-waters in 2020 possibly indicate the effect of the OYC on the coastal Doto, although the contribution was not very large.

In 2020, the ^134^Cs concentrations in the OYC-affected waters (the OY–E/O–S- and OY–KW-waters) in the off-Doto region (mean: 1.1 mBq/L) were ~ 1/8–1/10 times those in the surface waters in the transient area to the side of the Japan islands in 2012^24^ and ~ 1/6 times those in the waters off the western American Coast in 2015^6^. The annual variation in ^134^Cs concentrations in the western Bering Sea (i.e., no notable concentration peak in 2017–2020)^9^ and the off-Doto region do not closely agree. This could be ascribed to the retention of ^134^Cs within the marginal Bering Sea and an annual change in the current paths in the upper EKC areas. Additionally, the disagreement in ^134^Cs concentrations in the ESC/OSW and ICW in the southwestern Sea of Okhotsk and the off-Doto region (Fig. 2e) reflects the time lag in the transport of ^134^Cs after the entry of the EKC along the Kuril Islands (~ 2 years)^21,25^.

In contrast to the significant dilution of the EKC caused by current mixing (maximum fraction of the upper EKC waters in the off-Doto region: ~ 0.4)^10^, the ^134^Cs concentrations in the OYC-affected waters in 2020 exhibited a small change (~ 1/2–1 times) in the EKC area. Additionally, in 2020, the concentrations in the S-waters (Fig. 3) were slightly lower than those in the OYC-affected waters. Therefore, the smaller decrease in the concentrations in the off-Doto region can be ascribed to the mixing of the ^134^Cs-contaminated currents (i.e., mixing of the SWC and ESC with the coastal waters and the KWC with the offshore off-Doto waters), which differs from the case of the dispersion-dominated areas (e.g., the western American Coast side to western Bering Sea).

**Figure 4:**
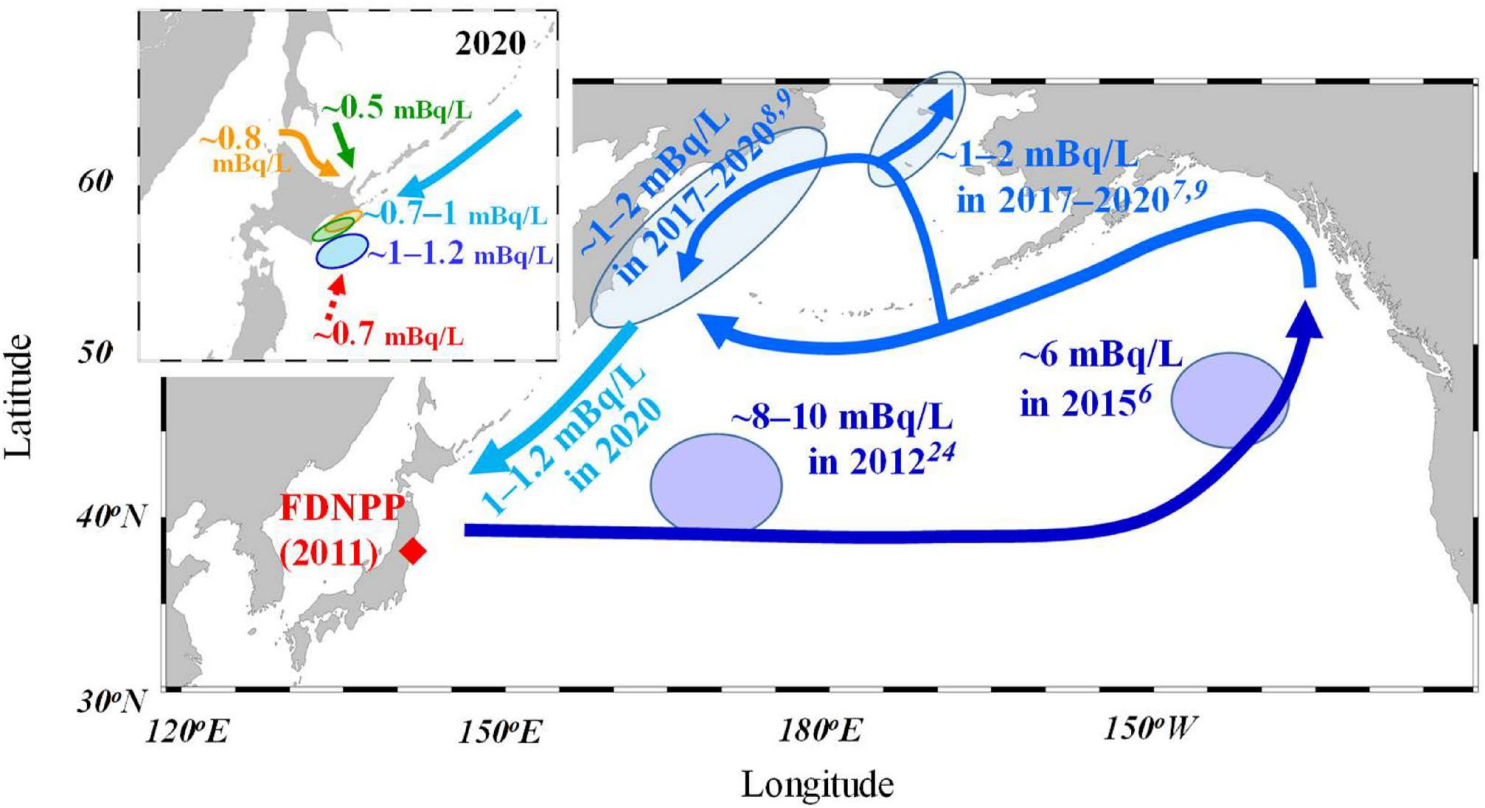
A schematic image illustrating the transport of ^134^Cs in the northern North Pacific Ocean, including the timescale, after the 2011 FDNPP accident^6–10,24^ and in the off-Doto region in 2020 (details in text and each reference).

The transport patterns of ^134^Cs in the northern North Pacific Ocean and the off-Doto region are presented schematically in Fig. 4:(i) ^134^Cs transport-dominated process: After widespread radioactive deposition^2,3^, a core highly contaminated with ^134^Cs was observed in the side of the Japan islands along the Kuroshio–Oyashio transition area (8–10 mBq/L) in 2012^24^ and off the western American Coast (~ 6 mBq/L) in 2015^6^.(ii) Dispersion process: Owing to the lateral and downward dispersions, ^134^Cs concentrations steeply decreased to 1–2 mBq/L in 2017–2020^7–9^ before reaching the Bering Sea and within it.(iii) Retention and circulation processes: High ^134^Cs concentrations (1–2 mBq/L) were continually recorded in the western Bering Sea in 2017–2020^9^, indicating the long residence time of ^134^Cs.(iv) Current mixing process: In 2020, the OYC highly contaminated with ^134^Cs had partially arrived the off-Doto region, showing a small decrease in the concentrations by mixing of other subarctic (e.g., ESC/OSW) and subtropical currents less contaminated with ^134^Cs.

The spatiotemporal distributions of radiocesium, particularly those of ^134^Cs, investigated in this study can provide a basis for predicting the transport, dispersion, and mixing patterns of soluble contaminants in the northern North Pacific Ocean, over a time.

## Materials and methods

The locations of the seawater sampling sites are shown in Table S1. We collected 41 seawater samples (~ 120 L each; ~ 100 L and ~ 20 L for radiocesium and ^228^Ra measurements, respectively) from the sea surface (depths of 0–2 m) in and around the off-Doto region between September 2020 and January 2022 during expeditions of the R/Vs *Hokko Maru*, *Wakataka Maru*, and *Soyo Maru*.

The chemical procedures for collecting radiocesium and ^228^Ra from seawater samples are detailed elsewhere^26,27^. ^134^Cs and ^137^Cs were separated quantitatively via co-precipitation by adding 1.04 g of CsCl and 16.0 g of ammonium phosphomolybdate (AMP) to ~ 80–100 L aliquots of unfiltered seawater samples. Subsequently, after adjusting the pH to 1 by adding another ~ 20 L aliquot of seawater, a Ba carrier minimally contaminated with radium was added, and BaSO_4_ was precipitated with the radium isotopes. The chemical yields were 90–93% for cesium isotopes and 92–100% for radium isotopes, based on the yields of the AMP/Cs (with a 95% mean removal yield of radiocesium from seawater during AMP treatment) and BaSO_4_ fractions, respectively.

Low-background γ-spectrometry was performed on all AMP/Cs and BaSO_4_ samples using Ge-detectors which were installed in the Ogoya Underground Laboratory, Japan^28^ and were completely shielded using ^210^Pb-free old lead. The spectrometry was performed for ~ 7 (radiocesium) or ~ 3 (radium) counting days. The ^134^Cs (605 keV) and ^137^Cs (662 keV) concentrations in the AMP/Cs fractions were calibrated using an AMP/Cs mock-up sample with known concentrations of ^134^Cs and ^137^Cs. The ^226^Ra (^214^Pb; 295 and 352 keV) concentrations were calibrated using a mock-up sample with approximately the same chemical composition as that of the water samples, including the uranium standard issued by New Brunswick Laboratory, U.S.A. (NBL-42–1) and ^228^Ra (^228^Ac; 338 and 911 keV), based on the detection efficiency curve, respectively. The analytical precision, based on 1σ statistics, was approximately 15–40% for ^134^Cs, 1–2% for ^137^Cs, 3–8% for ^226^Ra, and 10–30% for ^228^Ra.

## Supplementary Information

Supplementary Information.

## Data Availability

The datasets used and/or analyzed during the current study are available from the corresponding author on reasonable request.
